# Understanding the impact of the COVID-19 pandemic and its control measures on women and children: a Zimbabwean case study

**DOI:** 10.3389/fpubh.2025.1659703

**Published:** 2025-11-06

**Authors:** Tinotenda Taruvinga, Rudo S. Chingono, Mandikudza Tembo, Ioana D. Olaru, Kenneth Masiye, Claudius Madanhire, Sharon Munhenzva, Sibusisiwe Sibanda, Lyton Mafuva, Natasha O’Sullivan, Abdinasir Y. Osman, Kevin Deane, Tsitsi Bandason, Manes Munyanyi, Annamercy C. Makoni, Solwayo Ngwenya, Karen Webb, Theonevus T. Chinyanga, Rashida A. Ferrand, Justin Dixon, Katharina Kranzer, David McCoy

**Affiliations:** 1 The Health Research Unit Zimbabwe (THRU-ZIM), Biomedical Research & Training Institute (BRTI), Harare, Zimbabwe; 2 Department of Global Health and Development, London School of Hygiene and Tropical Medicine (LSHTM), London, United Kingdom; 3 Clinical Research Department, London School of Hygiene and Tropical Medicine, London, United Kingdom; 4 Organisation for Public Health Interventions and Development (OPHID), Harare, Zimbabwe; 5 Department of Public Health and Primary Care, Queen Mary University of London (QMUL), London, United Kingdom; 6 MRC Epidemiology Unit, University of Cambridge, Cambridge, United Kingdom; 7 Pathobiology and Population Science, Royal Veterinary College, London, United Kingdom; 8 Ministry of Health, Mogadishu, Somalia; 9 Ministry of Health and Childcare, Harare, Zimbabwe; 10 Harare City Council, Harare, Zimbabwe; 11 Mpilo Central Hospital, Bulawayo, Zimbabwe; 12 Department of School of Medicine, National University of Science and Technology (NUST), Bulawayo, Zimbabwe; 13 Division of Infectious Diseases and Tropical Medicine, LMU University Hospital LMU, Munich, Germany; 14 International Institute for Global Health (UNU-IIGH), United Nations University, Tokyo, Japan

**Keywords:** impact, COVID-19 pandemic, women and children, case study, Zimbabwe

## Abstract

The Coronavirus Disease 2019 (COVID-19) posed significant health policy challenges, particularly for low-income countries, where policymakers faced both direct threats from the virus and social and economic harm owing to lockdown measures. We present a holistic contextualized case study of the direct and indirect impacts on women and children, highlighting disparities across socioeconomic, age, and gender groups. We utilized multiple data sources, including primary and secondary data from 28 in-depth interviews, six focus group discussions, and 40 household interviews, as well as data from government reports, District Health Information Software version 2 (DHIS2), and published research. A conceptual framework was devised to hypothesize causal pathways and guide the analysis of results. The findings indicate that the pandemic not only had direct effects, on morbidity and mortality, but also more severe indirect impacts, including job losses and limited access to healthcare, including maternal and child healthcare services, due to measures put in place to control it, which were exacerbated by well-known but inadequately considered preexisting political and economic challenges. The most severe indirect effects on healthcare services availability and wider livelihoods fell on the poorest segments of society, further widening the age and gender inequalities. Policymakers faced significant challenges in managing the direct and indirect harm of COVID-19, including short- and long-term effects and their unequal distribution across society. We conclude that the indirect effects of COVID-19 were at least as harmful, if not more so, than the direct impacts, especially for women and children. In the future, it is highly recommended to establish specific protocols and guidance for maternal and child health service access, including strategies that reduce barriers to social support.

## Introduction

The Coronavirus Disease 2019 (COVID-19) pandemic presented all countries with major health challenges (1, 2). These included ensuring adequate epidemiological surveillance of COVID-19, implementing infection prevention and control (IPC) measures, providing care for COVID-19 and non-COVID-19 patients, and delivering COVID-19 vaccines. Low- and middle-income countries (LMICs) were also faced with having to implement ‘draconian’ lockdown measures (as characterized by the stringency index) (3) without having the resources to mitigate their social and economic harm (4) and the disruption caused to normal healthcare provision (5).

Importantly, countries had to consider how the harm associated with the pandemic would be unevenly distributed (1). For example, wealthier households were better able to withstand the psychological and emotional stresses of the lockdown than poorer households (6, 7). Additionally, there were reasons to think that women and girls would experience lockdown measures differently from men and boys (8). Prior evidence has shown that women have been significantly impacted socially, economically, and in terms of healthcare access and outcomes. Economically, women were 1.8 times more likely to lose their jobs than men (7, 9–11). Socially, in sub-Saharan Africa, the pandemic increased the burden of unpaid care work, which includes child care, cooking, and cleaning, leading to a higher job drop rate than males (12–14). Additionally, some studies have reported a significant negative impact on access to essential health services, including maternal and child healthcare (15–17). Finally, the early observation that infection fatality rates varied substantially across age groups raised challenges for policymakers in balancing the different needs of children and adults (15). However, there is still limited knowledge on the impact of the pandemic and the intersectional impacts on women and children in LMICs, including Zimbabwe.

This study aimed to present a holistic and contextualized case study of the direct and indirect health impacts of COVID-19 on women and children in Zimbabwe, with an assessment of their uneven distribution across socioeconomic, age, and gender groups. Zimbabwe’s long-standing political, historical, and socioeconomic challenges and fraught relations with Western nations make it a particularly relevant setting for such a study. While many aspects of the case study are place-specific, its implications extend beyond Zimbabwe, with lessons for global pandemic preparedness and equity-oriented policy. As is typical of case studies seeking to understand complex phenomena, we used different types of primary and secondary data from multiple sources (18) to produce an integrated chronological descriptive analysis of the COVID-19 epidemic, its policy responses, and its impact on maternal, child, and women’s health.

## Methods

### Study design and setting

We adopted a case study approach to present a descriptive analysis of the COVID-19 epidemic in Zimbabwe, including policy responses to the pandemic and its impact on health and healthcare. In this country case study, we performed a more detailed examination of the urban provinces of Harare and Bulawayo, using both qualitative and quantitative data collected concurrently. The two cities are major urban centers in Zimbabwe, with the former as the capital and the latter as the second-largest city.

### Data collection

To produce a chronological narrative account of policies related to COVID-19 from February 2020 to August 2021, using a policy review checklist we collected data from government policy documents and announcements, newspaper articles, social media, and the Oxford COVID-19 response tracker (OxCGRT), a project that collated data on COVID-19 control measures from countries across the world (19). The policy review checklist was basically an excel sheet which captured COVID-19 policy data. The tool captured the name of the policy document, the type of policy document, its date of publication, the content of the document, and its implications. Five research assistants completed the checklist, and it was reviewed by the study coordinator and the two Principal Investigators (PIs) to enhance rigor.

To present an account of the COVID-19 pandemic, we obtained official data on the number of COVID-19 tests performed, confirmed cases, and deaths from daily situational reports and the official Twitter (now X) handle of the Ministry of Health and Child Care (MoHCC). Given the limited disease surveillance and low testing rates, we also drew on data from other studies to assess COVID-19 transmission in Zimbabwe.

To describe the effects and impacts of COVID-19 and its control measures on maternal and child healthcare, we extracted routinely collected health service data on antenatal care (ANC) and growth monitoring of children and childhood vaccinations from District Health Information Software version 2 (DHIS2) for the period January 2016 to August 2021 in the Bulawayo and Harare metropolitan provinces. Harare, the capital city, and Bulawayo, the second largest city in the country, have estimated populations of 1,896,134 and 676,650, respectively, and together account for approximately 30% of the country’s recorded COVID-19 cases (20). Primary health services in the two cities are primarily administered by the city council, whereas tertiary and quaternary hospitals are administered by the central government. We selected 20 public primary health clinics in Harare and 18 clinics in Bulawayo to study the trends in healthcare provision and utilization.

We selected two child and two maternal services: growth monitoring (GM), child vaccination, antenatal care (ANC), and HIV testing of pregnant women. Several data items are routinely collected to monitor child vaccination services indicators, and we chose to analyze trends in the number of children aged 9–12 months who have received their final measles, mumps, rubella (MMR) vaccine, which is captured in the DHIS2 as ‘primary course complete’ (PCC). For maternal health services, we analyzed trends in the number of pregnant women attending a 4^th^ ANC visit (a proxy indicator of adequate ANC visits according to the WHO), as well as the number of women who received an HIV test at their first ANC visit. However, data for the latter were only available from 2019 onwards.

We also collected primary qualitative data between April 2021 and December 2021 in Harare and Bulawayo. We conducted face-to-face 28 in-depth interviews (IDIs) of key informants drawn from community-based organizations (CBOs; *n* = 5), city health managers (*n* = 5 Harare, *n* = 3 Bulawayo), national program managers and policymakers (*n* = 5), and healthcare workers (*n* = 3 Harare, *n* = 7 Bulawayo). We further conducted two focus group discussions (FGDs) with healthcare workers from selected primary health facilities in Harare (*n* = 12) and Bulawayo (*n* = 11) and four FGDs with members of the public (two in Harare with nine and 10 participants, and two in Bulawayo with 11 and 13 participants). In addition, we conducted 40 household interviews evenly distributed across the three economic strata (*n* = 20 Harare and *n* = 20 Bulawayo), four households all from the higher income bracket declined to be interviewed. We purposively identified economically stratified residential areas and then randomly selected households within these strata. Topic guides were used for all qualitative interviews, with interviews lasting an hour and FGDs lasting an average of 2 h. Primary qualitative data were collected by five experienced and trained (three research assistants, one study coordinator, and one principal investigator) fluent in English, Shona, and Ndebele. Separate topic guides were used for different informant groups, including questions about their experiences and views on COVID-19 and its control measures as well as about health services and treatment access during the pandemic. The focus was on understanding the impact of the COVID-19 control measures on maternal and child health.

Finally, for policy documents, we drew data from the selected published literature to describe the historical and socioeconomic context of the pandemic and added further information about the impact of COVID-19 on maternal and child health in Zimbabwe. We identified this literature from the PubMed and Google Scholar databases for papers published up to January 2023 using the search terms ‘maternal and child health’, ‘COVID-19,’ and ‘Africa’ or ‘Zimbabwe’.

**Figure 1:**
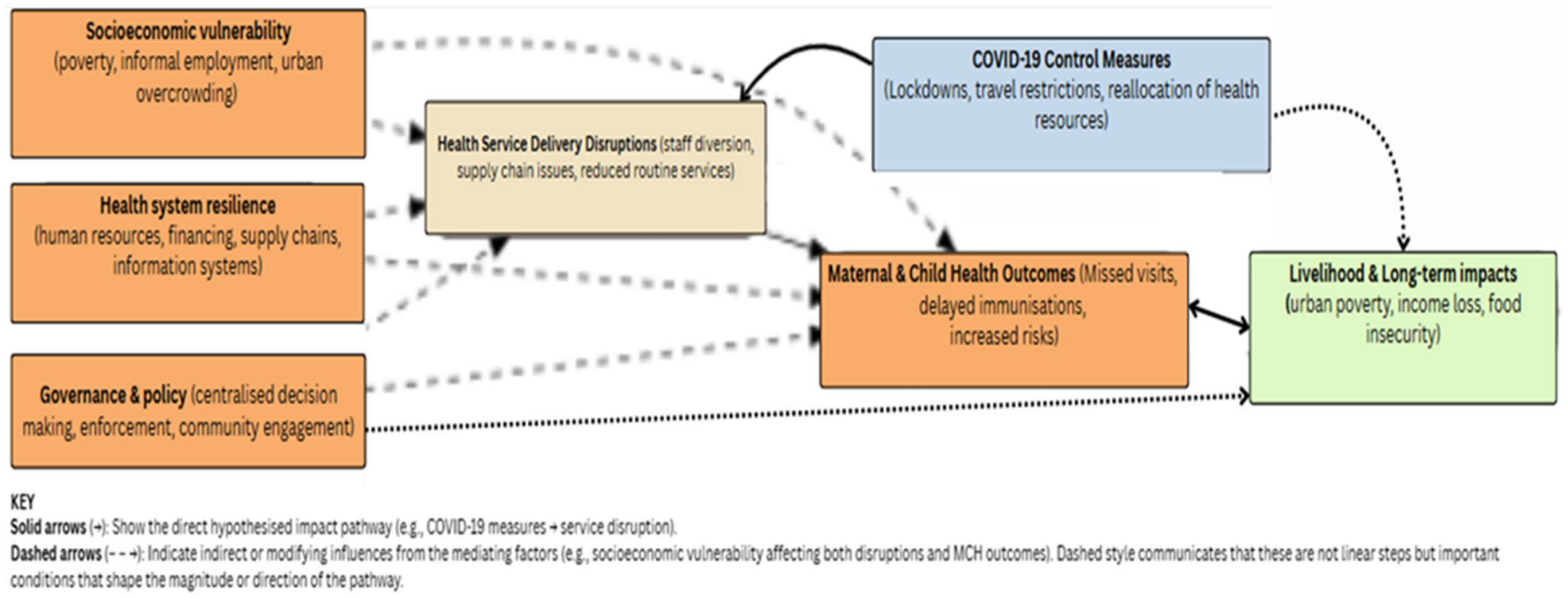
Conceptual framework for analysis.

To guide our analysis, we developed a conceptual framework, (see Figure 1) that broadly captures the hypothesized impacts of pandemic control measures, including lockdowns, travel restrictions, and the reallocation of healthcare resources. We hypothesized that, in addition to disrupting transmission as intended, these would also disrupt health service access and delivery, in turn affecting the utilization and outcomes of maternal and child health (MCH) services. Mediating and modifying factors interacting to produce observed impacts include socioeconomic vulnerability (including poverty, dependence on informal employment, and urban overcrowding), governance and policy processes (such as centralized decision-making, enforcement strategies, and community engagement), and health system resilience (encompassing human resources, financing, supply chains, and information systems). The solid arrows in the model depict the direct hypothesized causal pathway; the dashed arrows represent non-linear mediating and modifying dynamics unpredictably shaping the magnitude and directionality of effects.

### Data management and analysis

Changes over time in the four indicators of healthcare utilization are shown graphically for each clinic, stratified by city, with trend lines using loess smoothing and 95% confidence intervals. We used interrupted time series models January 2016 to the end of 2019. The ratios were color-coded using shades of orange to signify counts lower than the pre-COVID-19 average and shades of green for higher counts. Some clinics had missing values for some months, especially in 2021, and these are colored gray. These missing values included a combination of instances where there was no service uptake (e.g., the clinic was open but there was no service utilization, or the clinic was shut) and when there was service uptake, data were not recorded or uploaded onto the DHIS2. In the pre–COVID-19 with generalized least squares to assess monthly trends, accounting for seasonality and autocorrelation, and estimated immediate and slope changes at the onset of the COVID-19 lockdown. Clinic-level changes in healthcare utilization are shown as ratios computed by dividing monthly counts by the clinic-specific monthly average for the period from COVID-19 period, data were missing for 6 months in 6 clinics. For each missing month, values were imputed by averaging the observations from the preceding and following months. Data for HIV bookings were not reported to DHIS2 before January 2019 in Bulawayo and February 2019 in Harare and were not analyzed.

All interviews and FGDs were recorded digitally, transcribed verbatim, and transferred to NVivo14 software for analysis. In the field, notes were taken and interview summaries were prepared daily. A codebook was developed through a review of all transcripts by three researchers, who contributed to the development of a thematic framework of codes through consensus. Thematic analysis was used to identify patterns and themes in the data, with some codes determined beforehand and others emerging during coding.

All data sources (qualitative, quantitative and policy data) were triangulated at the interpretation stage to trace policy events and assess the impact of the COVID-19 pandemic and its control measures on the provision and use of healthcare services for women and children.

## Findings

### The context of Zimbabwe’s COVID-19 epidemic

Zimbabwe is a low-income landlocked country with approximately 15 million people, 42% of the population under the age of 15 (20). Approximately 71% of the population lives below the international poverty line of $1.90 per day (21) leading to poor health indicators. Over 80% of income earners operate in the informal sector (22) which contributes to over 60% of the gross domestic product (GDP) (22, 23). One in four of the urban population (about 1.25 million people) lives in settlements with poor water and sanitation infrastructure, as defined by UNICEF (24).

The country experienced severe political and economic challenges for the three decades before the COVID-19 pandemic. An unsustainable external debt burden and structural adjustment program resulted in a severe contraction in public expenditure in the 1990s, which weakened most public services (25). The turn of the millennium saw that the country entered a period of protracted political turmoil and the imposition of targeted sanctions by several European countries (25, 26) further undermining the economy. Just before the pandemic, Zimbabwe’s GDP declined by 11.3% in 2019 compared to 2018, and continued to contract in 2020 (27).

Although Zimbabwe’s health system was once considered among the best in Africa (28) it deteriorated due to reductions in public spending, out-migration of health professionals, mismanagement, and industrial action (29, 30). By 2021, the doctor and nurse-person ratios had deteriorated to 1:12,000 and 0.39:1,000, respectively, far below the World Health Organization’s (WHO) recommended minimum threshold density of 2.28 doctors, nurses, and midwives per 1,000 people in developing countries (31). Today, there are high levels of out-of-pocket payments and donor dependency (17). The maternal mortality rate was 458 per 100,000 live births (32) and the child mortality rate was 55 per 1,000 live births in 2020 (33).

**Figure 2:**
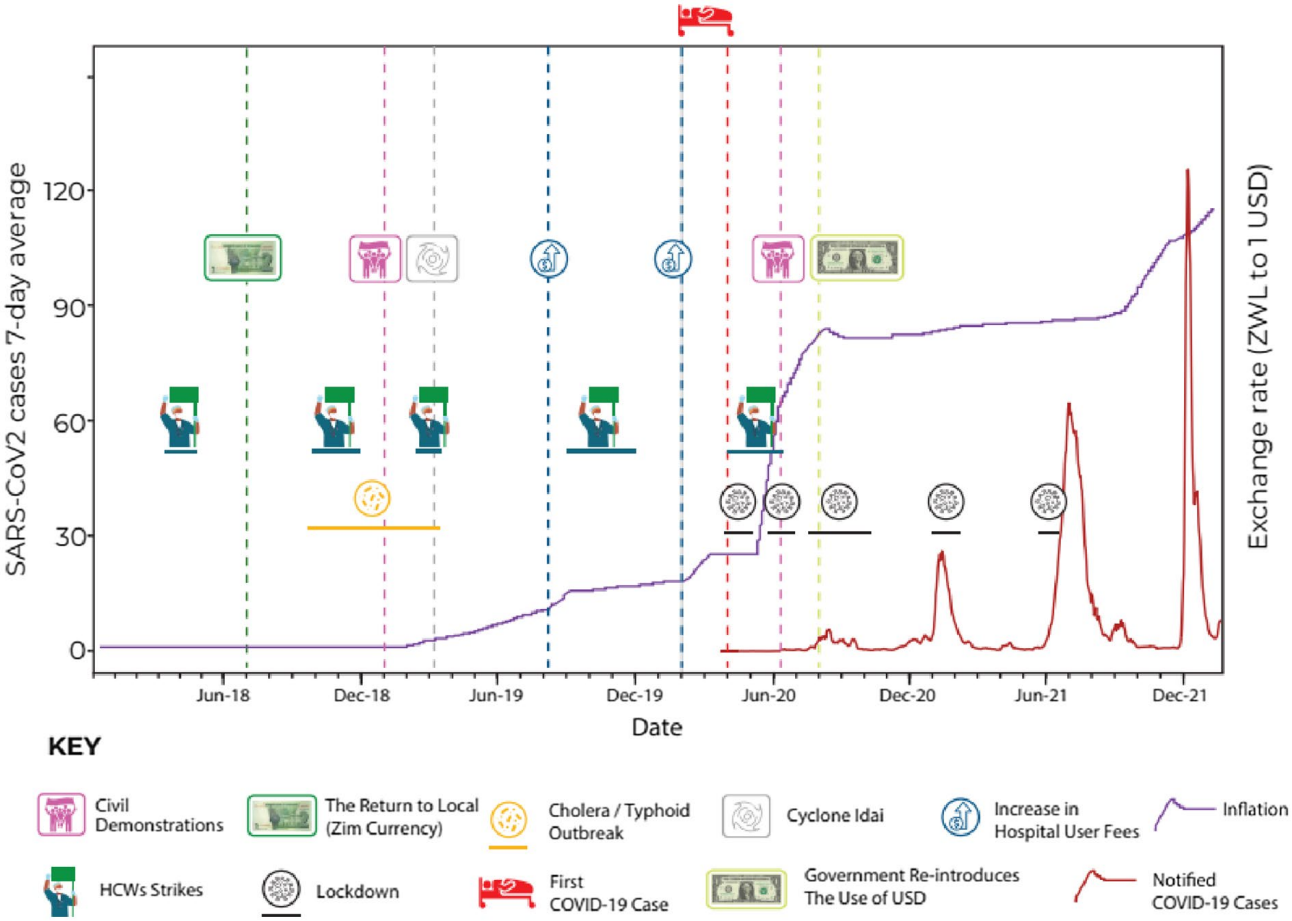
Timeline of key events concerning the period between January 2018 to December 2021.

Zimbabwe faced social, economic, and political challenges that impacted the country’s health system long before the COVID-19 pandemic. In addition to this chronic deterioration of the health system, there were a series of acute crises during the run-up to the pandemic (see Figure 2). This included doctors and nurses engaging in intermittent industrial action (30), typhoid and cholera outbreaks in 2018, a major natural disaster (cyclone Idai) (22) in 2019, and an acute worsening of inflation since April 2020, causing shortages of fuel and water.

The precarious state of the health system during the pandemic was noted in the qualitative data (Figure 2).

“*Most of our stream mates have gone to the UK where there are better jobs. Others are in the process of applying; we are all trying to leave for greener pastures (laughs). Those of us who stay are mostly working out of duty…people are disgruntled, and this can be sometimes seen through our attitudes and, the strikes.”* (Nurse, HCW FGD, Bulawayo).

“*Unfortunately, in Bulawayo when COVID-19 was declared and it hit us, we were facing water challenges and other related things.”* (Doctor, KII, Bulawayo).

### Evolution of the COVID-19 epidemic and control measures

#### January to May 2020

Following WHO’s call to prepare for the containment of COVID-19 in January 2020, the government of Zimbabwe quickly started developing a National Preparedness and Response (NPR) Plan and established an Interagency Coordination on Health and Epidemic Preparedness and Response Task Force (34) screening for Severe Acute Respiratory Syndrom Coronavirus 2 (SARS-CoV-2) infections at major entry points was quickly implemented for all incoming individuals. In February and March, the private sector, civil society, academia, professional associations, technical and donor partners, and the media participated in a series of inter-ministerial meetings on COVID-19.

The NPR Plan was informed by a Preparedness and Response Plan for Pandemic Influenza A H1N1 formulated in 2009, recent simulation exercises, a comprehensive review of the 2018 cholera outbreak, and a rapid assessment of the state of preparedness at major points of entry into the country and major health facilities in February. The Plan was aligned with the WHO’s COVID-19 Strategic Preparedness and Response Plan and was finalized in March.

On March 17^th^, a state of national emergency was declared prior to any detectable surge in SARS-CoV-2 infections. The first case of COVID-19 was only reported on 20^th^ March in an individual who had recently returned from the United Kingdom. Two additional cases imported from the United States were documented 2 days later. The first recorded fatality occurred on 23^rd^ March (35), the same day the Civil Protection (Declaration of State of Disaster: Rural and Urban Areas of Zimbabwe; COVID-19) notice was issued (36).

A Public Health Act (COVID-19 Containment and Treatment) was also enacted on the 24^th^ of March, mandating compulsory testing of individuals suspected of infection and quarantine of those who tested positive (37). Subsequently, a Statutory Instrument (SI) was introduced, imposing a 21-day lockdown from the 28^th^ of March during which time public gatherings of more than two people were prohibited. While ‘essential services’ were allowed to operate, retail outlets were allowed to open only during specific hours with mandatory screening and hand sanitization at entrances. Although schools and universities were initially slated to remain open until the end of the term, a later announcement mandated the closure of all educational institutions from 24^th^ March 24, 2020. The sale of alcohol was banned, and stockpiling of medical supplies and food was explicitly prohibited, with strict penalties for violations.

During this phase, the government designated certain health facilities as quarantine and isolation centers, adopted and adapted the WHO’s COVID-19 case management guidelines (38), organized training for health workers, and upgraded several government and private hospitals. Surveillance teams were created to facilitate case investigation and contact tracing. Over 4,000 health sector posts were unfrozen, and an additional 200 new medical posts were created and funded through the reallocation of funds (39) from other departments and the proceeds of the 2% intermediate money transfer tax, which is normally used for social protection and capital development projects. However, despite efforts to strengthen the health system’s capacity to respond to COVID-19, the shortage of PPE and inadequate pay led many healthcare workers to feel vulnerable and undervalued (40) and precipitated a fresh round of industrial action beginning on 25^th^ March with a nurse’s strike (41).

*“An association representing the City of Harare nurses went around to check if there was adequate PPE…After they observed that there was nothing that’s when they called for the nursing staff or for the healthcare worker to stop going to work, ‘tools down guys there is no PPE until proper adequate PPE is in place’. So, you find that most nurses of the city of Harare went home and stayed…The situation was also aggravated by the fact that we were being poorly remunerated, we were not getting our salaries on time.”* (Nurse, HCW, FGD, Harare).

The lockdown measures were eventually extended for an additional 4 weeks, until May 17th. However, starting on May 3rd, the commercial and industrial sectors were permitted to operate between 0800 and 1,500, provided they adhered to IPC measures. During this period, several statutory instruments were issued to modify or extend initial lockdown regulations. For instance, on April 3rd, it became mandatory to wear facemasks in public, and transportation services were required to check the temperatures of passengers boarding their vehicles and implement disinfection protocols. Initially, residents returning abroad were isolated from the designated facilities. However, on May 4th, all international travel was banned. Land border crossings at Beitbridge (bordering South Africa) and Plumtree (bordering Botswana) were also closed and internal road travel was restricted, necessitating exemption letters at checkpoints. The number of reported cases remained low throughout the study. When the lockdown was lifted on May 17th, Zimbabwe recorded only 46 cases and six deaths. However, owing to the limited public sector testing capacity and the prohibitive costs of private testing for most individuals, the actual number of cases during this time is uncertain. Furthermore, there were no formal or informal reports of any surges in hospital admissions or mortality during the study period.

#### June 2020–November 2020

After the first lockdown was lifted, the number of recorded cases remained low for approximately 8 weeks. Despite this, the government imposed a two-week lockdown period, from June 20 to July 4. This followed extensive civil society protests following the arrest of the Health Minister on allegations of graft. It is widely believed that lockdown was used to suppress political opposition rather than the epidemic (42, 43), with many protesters arrested for contravening new COVID-19 regulations.

However, when the number of cases began to rise, the government introduced a third lockdown on July 24^th^. Night-time curfews (from 6 p.m. to 6 a.m.) and travel restrictions were implemented. However, essential services continued to operate as usual and as non-essential businesses, and low-risk sports were allowed to operate from 9 a.m. to 3 p.m. Restaurants, hotels, and other tourism services were allowed to operate at 50% capacity, and schools were kept open until July 30^th^ to allow the completion of national examinations. Although the increase in the number of cases was partly due to increased testing capacity (44) there was some evidence of a real increase in the incidence of infection with rising COVID-19 mortality and hospitalizations (45) including some high-profile fatalities.

The third lockdown continued until September, but with the progressive easing of restrictions, case numbers started falling. Government quarantine centers were phased out in August and replaced with self-isolation in private premises, and a phased opening of schools commenced on the 8^th^ of September and was completed in November. The sale of alcohol for off-premises consumption was allowed on September 30, and international airports began operating again in October. Initially, only Zimbabwean residents were allowed to return (provided they tested negative and were self-quarantined at home for 14 days); entry was later allowed for foreign travelers. In December, the land ports of entry opened.

#### December 2020–April 2021

In late November 2020, a new wave of cases emerged, driven by the Beta variant (46). This coincided with the reopening of schools where large outbreaks have been reported (47). As numbers rose and with several high-profile deaths, including those of five cabinet ministers (48), the government introduced a fourth national lockdown for 30-days on January 3, which was extended to February 16 and then again to February 28. The government closed schools, prohibited gatherings, reintroduced curfews, and stopped intercity travels. Mask wearing, hand sanitisation/washing, and temperature checks were mandatory for the public. As the country started recording a reduced number of cases, lockdown measures were eased in March, intercity travel resumed, and schools were reopened in a phased manner. April saw a relaxation of most remaining IPC measures, although international travel to and from countries such as India that had reported the Delta variant were not permitted, while travelers from other countries were required to present a negative COVID-19 test result and self-quarantine at home or in designated quarantine centers for 10 days (49).

During this period, the country also began a COVID-19 vaccination program, with the target of vaccinating 10 million people by December 2021 (50). The first phase prioritized frontline healthcare workers, the above 65 years, and those with chronic medical conditions (51). The second phase, which began in March 2021, extended coverage to uniform forces, all civil servants, and those offering essential services in the private sector. The last phase was introduced in July and extended coverage initially to everyone over 18 years, then to anyone over 16 years, and finally to anyone over 12 years (52).

#### May 2021–September 2021

In mid-May, a new (Delta) variant was detected in the Kwekwe district, Midlands Province. A new SI was introduced to allow for a localized lockdown of the Kwekwe District and in two other districts (Hurungwe and Kariba) (53). Eventually on June 27th, due to rising cases, a fifth full lockdown was implemented across the country. However, despite the increase in cases, the government came under pressure from parents and opened schools in the first week of August, 2021. This was followed on August 10 by the reopening of some social activities; for example, vaccinated congregants were allowed to attend church services.

By the end of September, 3,051,371 first and 2,211,880 s second vaccine doses were administered nationally, translating to a national coverage of 35.7 and 25.8%, respectively (54). At approximately the same time, the Global Fund pledged USD 75–150 million (55) to mitigate the impact of the COVID-19 pandemic on HIV, tuberculosis, and malaria. From September onwards, the country gradually moved into the post-pandemic phase even though there was no official declaration of the pandemic. However, the government emphasized more on ‘living with the new normal.’

### Policy implementation

According to our key informants, a notable feature of the response to COVID-19 was that policies were implemented in a centralized and top-down manner with little bottom-up and contextualized input.

*“There was centralisation of decision making and policy formulation …. everything was moved to the centre …and ours was just to implement. Then we had a situation where there was a take-over … of local authority institutions by central government. It had its downside and upside. But the formulation of that policy, unfortunately, we did not input in.”* (Doctor, KII, Bulawayo).

*“Let us not take that one size fits all or… maybe some African countries are doing this, you just follow suit and do this …. We should design our measures to suit our people, to suit our needs and to suit our country and our resources.”* (Nurse in charge, IDI, Harare).

*“I still insist that we must not have a lockdown that stretches from Zambezi to Limpopo, that is being the same. Let us modify it and say we are now in Gweru. How do we make this lockdown work? When we get to Chirundu, it must not look like the one in Chiredzi. There must be that distinction. I feel that with the localised input and the modifications we make, the lockdowns will be more effective. Such that it will allow us to even open the economy in one part of the country whilst the other part is under lockdown.”* (Doctor, KII, Bulawayo).

Informants also noted that public health communication (mainly conducted through the Zimbabwean broadcasting cooperation’s television and radio channels) was not fully effective, and that misinformation and disinformation on various social media platforms contributed to poor compliance with COVID-19 control measures, despite rapid response teams being established to respond swiftly to rumors and false information, albeit with limited capacities to deal with all rumors due to a shortage of human and material resources. Others have indicated that many people feel overloaded with conflicting information (40).

*“What was our challenge was people did not have information. So, you would just see a uniformed person come to you to hit you and you do not have information, especially at the beginning. People were just being hit for COVID, but if you would have educated people … ‘this is COVID, people are affected this way, we need to prevent using these methods’ … I think we are an educated nation that can actually follow through and take instructions”* (CBO representative, KII, Bulawayo).

*“We communicated, we talked, we tried to send messages, but I do not think it was effective because people had their own expectations, and they had their own other sources of information. And they did not trust the figures that were coming out of the Ministry of Health and situational reports, however, localised or frequent, people still did not feel that they were in as much danger as it was being portrayed.”* (Doctor, KII, Bulawayo).

Another theme that emerged from our primary data was the impracticality and impossibility of implementing social distancing measures, especially in high-density and overcrowded informal settlements with inadequate water and sanitation infrastructure (56). Other measures such as the use of hand sanitizers were unaffordable. According to one study in Harare and Mashonaland East, between 18 and 53% of healthcare workers reported a lack of soap, water, and masks between June 2020 to the middle of 2021 (57). Other studies have similarly described how communities cannot comply with social distancing measures because of the need to secure food, water, and income (40).

*“We expect the government not to implement water cuts and to improve water supply during such a time because we are having a difficult time, social distance is not possible [in the queues], we are at risk of contracting COVID-19”* (Participant, household IDI, Harare).

*“It was impossible to comply with the social distancing measure, at a time when there were food shortages and one had to stand in long queues. I still remember there was a time I went to a queue to get mealie-meal at Malbereign. The soldiers had to come and space out the people.”* (Participant, household IDI, Harare).

The inability to monitor and enforce travel restrictions was another cause of poor compliance with the lockdown measures. Despite the deployment of the police and the army, resources were insufficient to enforce quarantine and ensure adherence to travel restrictions. For example, despite major points of entry being manned by armed security, many people were able to enter the country illegally without being quarantined (58).

The relative ineffectiveness of lockdown measures may also be deduced from seroprevalence studies, which found that, by the end of March 2021, a high proportion of the population was infected (59). A systematic review of seroprevalence studies in Africa between January 2020 and December 2021 also estimated that the seroprevalence in Southern Africa was 56.1% (95% CI 44.6–66.9%) by the third quarter of 2021, with lower rates in rural areas and high heterogeneity between countries (59). In a study of randomly selected households from three high-density communities in Harare, seroprevalence increased from 19% in November–December 2020 to 53% in February–April 2021 (58), suggesting high levels of community transmission during the second wave. A survey of healthcare workers as early as June 2020 reported that 39.1% had a history of COVID-19 symptoms and a SARS-CoV-2 seroprevalence of 8.9% (60). The reported data also indicated a less severe disease profile in Africa, with more asymptomatic cases than in other parts of the world (61).

Another feature of the policy response to COVID-19 was the government’s efforts to mitigate the social and economic harm of the lockdown. In May 2020, the government announced a COVID-19 Economic Recovery and Stimulus Package worth approximately 9% of the GDP to mitigate the lockdown’s social and economic impacts. This package included US$5 monthly income support for vulnerable households and fiscal support for sectors like manufacturing, agriculture, mining, and tourism (62). Temporary measures were also introduced to defer rent and mortgage payments. However, the income support was not fully implemented, with only 202,000 of the 1 million eligible households receiving it 4 months into the lockdown (23). A compensation scheme for civil servants who lost their lives on duty was similarly incomplete, but all civil servants were granted a US$75 monthly COVID-19 allowance for over 36 months, in addition to their regular salaries.

*“I think maybe what probably might have lacked…are the necessary support systems to make sure that we take care of the most vulnerable in society, for example, if we say there is no public transport that is going to be operating, we should then be able to provide transport for those that want to seek health services.”* (NGO representative, KII, Harare).

*“For an economy that is largely informal, if we then say we are closing everything else all the markets non-informal business and everything else, we should then put in place a system where we can then support those in the informal sector so that at the end of the day, they do not starve.”* (CBO representative, KII Bulawayo).

### Impact of COVID-19 control measures on maternal and child healthcare

**Figure 3:**
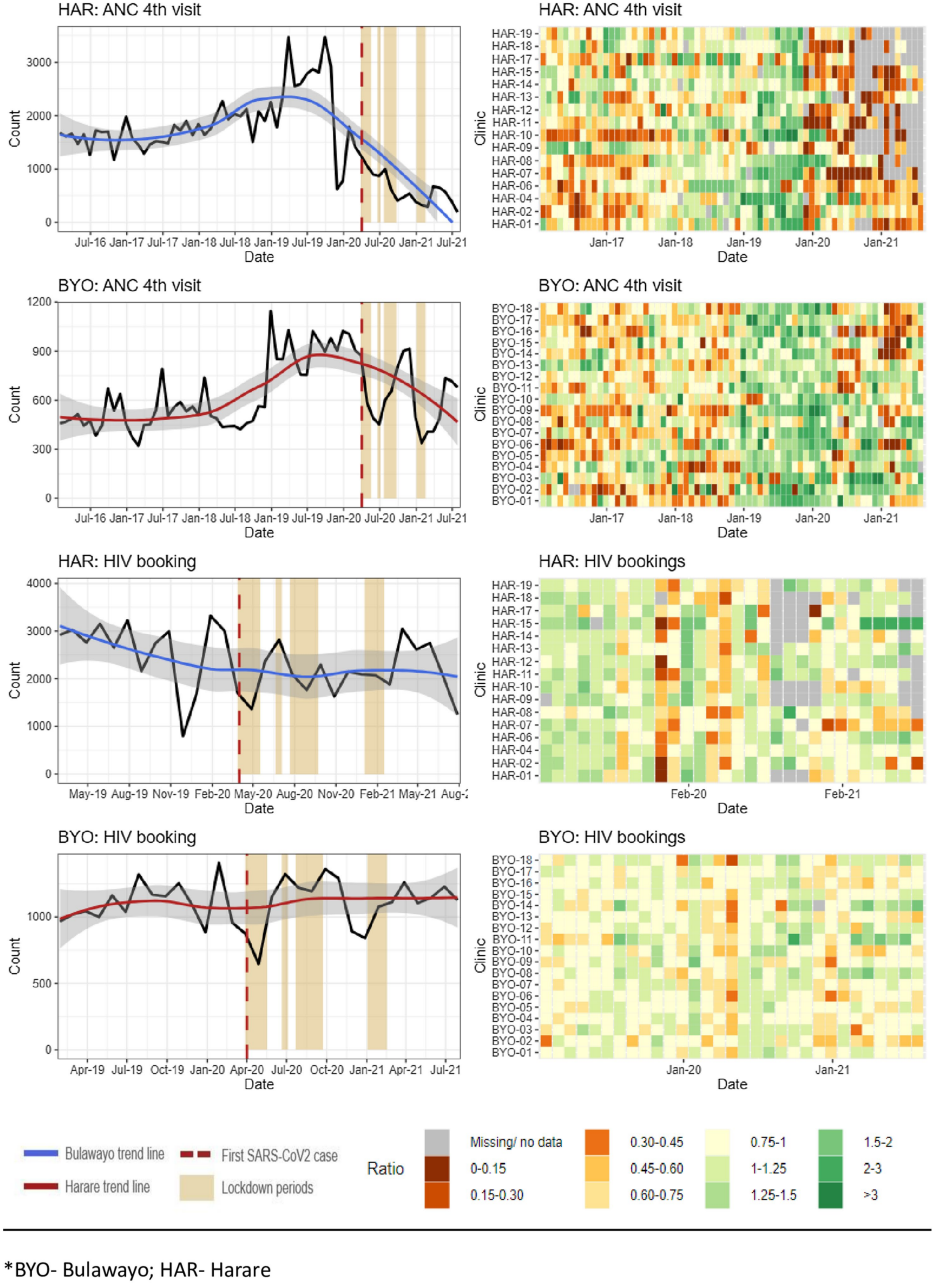
Source: Zimbabwe District Health Information Software Version 2 maternal indicators.

Our analysis of routinely collected data from July 2016 to July 2021 in Harare and Bulawayo found evidence of a reduction in healthcare utilization. Figure 3 presents data on the number of fourth antenatal care visits by pregnant women and the number of HIV tests conducted among pregnant women at their first antenatal visit.

The data show a reduction in the number of fourth ANC visits in both provinces, with a decrease of 29% in Harare and 34% Bulawayo following the COVID-19 lockdown. However, there was no observable reduction in the number of HIV tests among the women attending antenatal care (Supplementary Table 1).

Key informants explained that the HIV program, which is largely donor-funded and relatively well resourced, was better able to maintain their pre-COVID-19 level of service. In a study conducted in Harare, the number of pregnant women accessing HIV services remained the same, except for a slight decline during the first lockdown (5).

**Figure 4:**
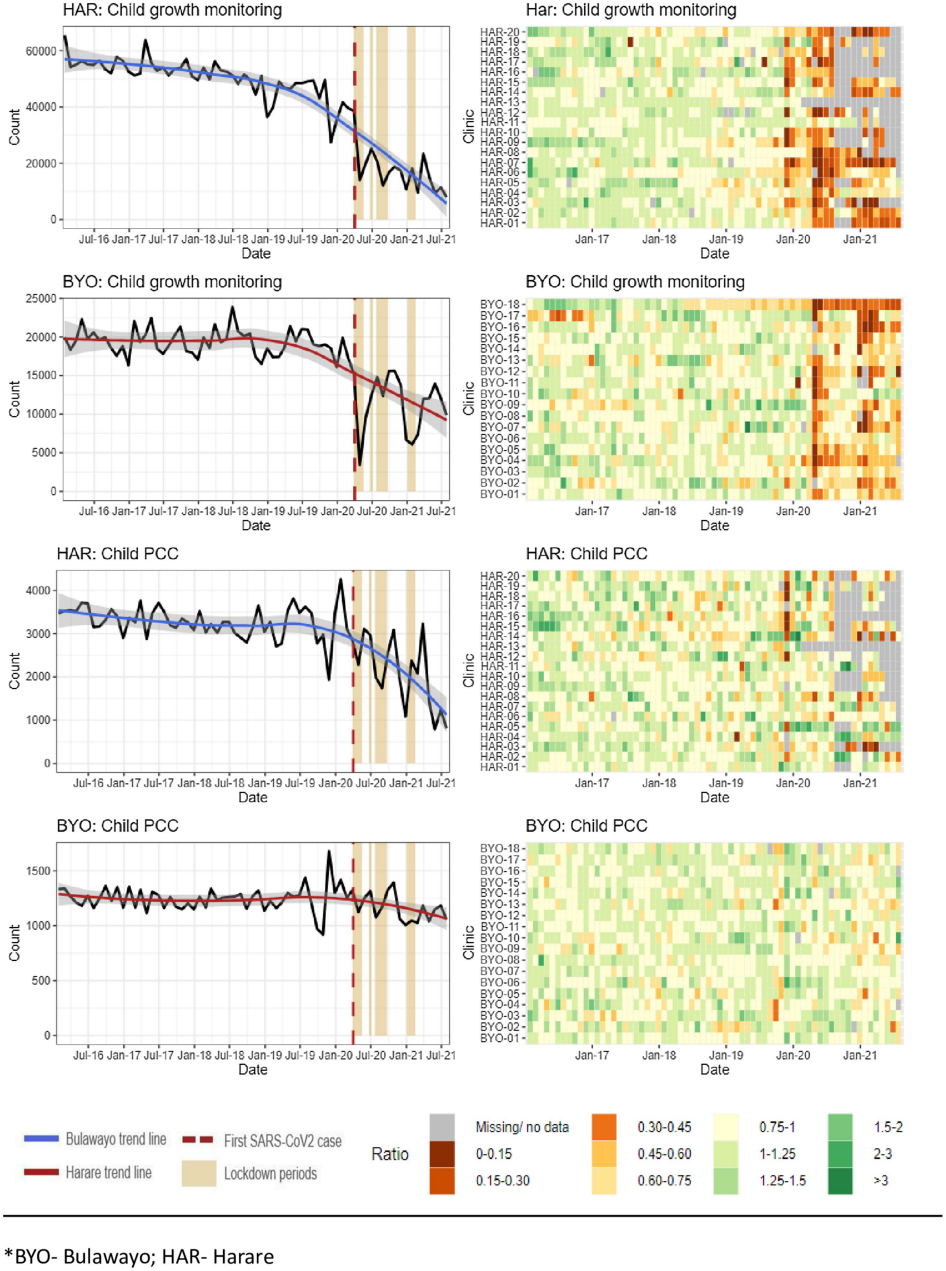
Source: Zimbabwe District Health Information Software Version 2 child indicators data.

Figure 4 presents the data on the number of GM visits by children under 5 years of age and the number of child PCCs. The data show that the number of GM visits decreased by more than 50% in both Harare and Bulawayo after the first COVID-19 lockdown (*p* < 0.001; Supplementary Table 1). The number of PCCs decreased substantially in Harare from March 2020 onwards, but not in Bulawayo.

Other studies also report that healthcare utilization declined in 2020 and 2021 compared to earlier years (63, 64). A study at Mpilo Hospital in Bulawayo, although the mean number of monthly deliveries reduced from 747 in the first quarter of 2020 to 681 in the second quarter, the overall reduction was not substantial (65).

Our qualitative data pointed to several reasons for the reduction in healthcare utilization. In some places, clinics were closed because the staff went sick because of COVID-19 (66) or because of industrial action. Some informants explained that healthcare utilization also dropped because health facilities were perceived as “hotspots” for COVID-19 transmission, especially those that had been repurposed for COVID-19 services.

*“People are not coming as they used to because staff tested positive and the community heard about it, and so maybe they are fearing that maybe when they come here, they might contract COVID-19”* (Nurse, IDI, Harare).

*“I have a young sister who was pregnant at the time… she had to go to Murehwa because we thought it was safer than here in Harare, …”* (Participant, Household IDI, Harare).

Police roadblocks and checkpoints were highlighted as barriers to health care because of fear and unwillingness to disclose one’s health status. Healthcare workers reported that it was difficult to access public transport because they were perceived to be at risk to others, especially when wearing a uniform.

*“Then people started to have difficulties in attending health centres, funerals, no hospital visits, this is the period from July to August. Whereby we are saying even HIV status disclosure on a roadblock. People were now forced to disclose… So, this was a forced disclosure…”* (NGO representative, KII, Bulawayo).

*“And other people ended up not even attempting to go …. we have other people who are on ARVs whom we know that have defaulted because they do not want to be harassed because you gave them a lift and you get there, they are asked to produce a card by the police, at times they would want to hide their status and not produce that card so end up not going to the hospital.”* (Nurse, HCW, FGD, Harare).

*“Our staff could not get transport to work, no one was willing to give them a lift as people considered them to be a high-risk group…We also had 6 staff members who tested positive and the news spread in the local communities and people were avoiding this facility for fear of getting the virus…”* (Nurse in charge, IDI, Harare).

Health services were especially disrupted by operating times being reduced from 07:30–17:30 to 08:00–14:00 and by capping the number of patients seen per day. In some instances, operating hours were reduced even further because healthcare workers struggled to get to work or had to leave early to comply with curfews.

“*Yes, at the clinic, they would serve a certain number of people per day, so that people do not get crowded there. So, if they reach the number of people that they wanted per day, some would be returned home and be advised to come the next day*.” (Participant, Household IDI, Harare).

The pandemic diverted resources and attention away from MCH services. Informants reported that in some clinics, GM was completely suspended to allow for the prioritization of other health services. Stockouts particularly affected immunization and family planning services (67).

*“Then in those findings, it was noticed that there was growth monitoring reduction. Then we also go to maternity health services whereby during the COVID era, we are saying only ANC bookings were done. No subsequent visits were done, limited monitoring of BP checks. If you remember we were no longer doing these BP checks, no physical examinations were done. Then om HIV and AIDS testing, the numbers started to reduce, viral load collection we stopped doing it, CD4 count was stopped, the updating of green books was also stopped.”* (Nurse, IDI, Bulawayo).

Access to healthcare was also restricted by some facilities implementing a policy of patients, including pregnant women, showing a negative SARS-CoV-2 test result before admission. This led to patients being turned away from the hospitals, and ambulances refused to transport patients.

*“Pregnant mothers were requested to have results for COVID-19 first before admission to a facility and the test price was very high some of the mothers could not afford it… and even the ambulance services would request you to produce results first before they carry you. If you did not have the results, they would test you at a fee. I remember it was USD 60 for the COVID-19 test before you get into an ambulance.”* (Nurse, HCW, FGD, Harare).

Some healthcare workers believe that an increase in the number of home deliveries without a skilled birth attendant resulted in an increased number of largely unreported maternal and neonatal deaths.

*“I do not have statistics, but as I was on night duty, you would see that some people did not come for their regular reviews, some would come already complicated which if we had seen them on time, we could have prevented those complications.”* (Nurse in charge, IDI, Bulawayo).

*“I know plenty who needed maternity services but could not get them…in fact, I know someone who even died. They wanted to give birth…but they were supposed to get COVID-19 tests before accessing health services…that process was costly and trying to find money took long and she died before she could even be given medical attention.”* (Participant, household IDI, Harare).

Other studies have reported similar findings in women and children struggling to access MCH services during the pandemic (15, 65). In Nyanga, Manicaland province, Nyashanu et al. describe transport problems, roadblocks, shortage of medication and personal protective equipment, and lack of routine healthcare services affected routine health access such as antiretroviral therapy (40). Chimhunya et al. viewed strikes by healthcare workers at Sally Mugabe Hospital in Harare as an indirect consequence of COVID-19, which caused a decline in neonatal admissions (64).

## Discussion

The COVID-19 pandemic occurred in Zimbabwe when the country experienced decades of economic decline and chronic deterioration of its health system (21). In the years immediately preceding the COVID-19 pandemic, the health system experienced intermittent industrial action by nurses and doctors, a series of natural disasters, and outbreaks of cholera, typhoid and measles (68). Although the government increased its budgets for healthcare and unfrozen healthcare worker posts to help cope with the pandemic (39), the fragility of the health system also made it likely that lockdown measures would further reduce both supply and demand for healthcare (69).

Given this background, Zimbabwe faced significant challenges during the emergence of COVID-19. Like many other countries, policymakers had to deal with a lack of knowledge regarding the virus’s natural history, virulence, and transmissibility (70). The high rates of hospitalization and mortality observed in countries such as China and Italy, along with the World Health Organization’s declaration of a Public Health Emergency of International Concern on January 30, 2020, prompted many nations to implement strict lockdown measures before experiencing a surge in COVID-19 cases. These decisions were based on the beliefs of public health specialists and policymakers that the negative impacts of lockdown measures would be less harmful than the consequences of the virus itself.

Early estimates of the infection fatality rate suggested that enforcing lockdowns, including halting significant social and economic activities, was justified. However, by the middle of 2020, noticeable differences in the incidence of cases and deaths had begun to emerge worldwide (61). The relatively low incidence of cases and deaths in parts of Africa was particularly notable (61), leading to several hypotheses regarding the underlying causes. Among these were factors such as the low prevalence of risk factors, such as obesity, lower infection rates, effective mitigation measures, youthful age structure, favorable warm weather, and preexisting immunity from previous coronavirus infections. As of the end of 2020, there were only 13,867 officially reported cases and 363 deaths. By the end of 2021, the number of deaths attributed to COVID-19 had increased to 5,004 (71). Although it is likely that there were many unrecorded deaths due to COVID-19, there was no period during which hospitals were overwhelmed by COVID-19 patients or when morgues struggled to manage a sudden increase in deaths, unlike in other countries. Furthermore, seroprevalence studies indicated that substantial transmission had occurred by the end of 2020 (58), suggesting that the relatively low mortality rates were likely not the result of effective prevention of transmission.

Reliable, timely, and context-specific data are crucial for managing infectious disease outbreaks and epidemics. In Zimbabwe, gaps in the health information system have resulted in a lack of timely, accurate, and detailed accounts of the pandemic and its direct impact. Throughout the pandemic, policymakers and public health specialists made educated guesses about how the epidemic evolved and its effects. The deficiencies in the country’s health information systems also hindered their ability to adequately assess the harmful and sometimes devastating impacts of the lockdown measures. Even now, estimates of the size and causes of the increase in all-cause mortality since the pandemic are unclear.

A key question emerging from this research is whether Zimbabwe adopted the right set of measures to effectively manage both the direct and indirect threats to health posed by COVID-19, including the harm associated with lockdown. Here, one must be wary of the benefits of hindsight and the lack of reliable and timely data available to policymakers and public health experts. However, given the preexisting high levels of poverty and food insecurity before the pandemic (22), economic stagnation, reduced household income, and rising prices for basic commodities associated by lockdowns were inevitably impactful. The inability of many households to generate adequate income during the lockdown had drastic and detrimental effects on access to food and basic amenities and was compounded by mounting loneliness, distress, and mental illness. Although the government created a support fund for poorer households, the budget was inadequate and many eligible households did not receive the promised support. Studies from other LMICs have shown similar findings (7, 8) and many countries are currently still experiencing the long-term consequences of the economic contraction and increased levels of indebtedness that occurred in 2020 and 2021 (72).

Similarly, a negative impact on an already fragile, under-resourced, and under-utilized health system was inevitable. The disproportionate impact on children was particularly significant. While COVID-19 itself was considerably less harmful to children, the lockdown measures had the opposite effect. These measures have led to the cessation or reduction of essential childcare services and increased the vulnerability of infants and young children to reduced food intake. Consequently, many children were deprived of crucial healthcare services because of measures that primarily benefited the adults.

Furthermore, lockdown measures were not feasible for large population segments. Many informants noted that safe and adequate social distancing is impractical in high-density areas and informal settlements. For many households, prolonged quarantine and social isolation were arguably unjustified, especially given the harmful effects of lockdown. Moreover, the lockdown measures implemented during COVID-19 involved significant restrictions on civil liberties, which should only be enforced under extreme and valid circumstances. The coercive powers invoked by governments across the globe in the name of public safety could not only be misused during a pandemic but could also lead to the establishment of permanent systems of surveillance and social control. For instance, in Zimbabwe, lockdown measures were used to suppress political protests and there were reports of security personnel abusing their power to engage in corruption and sexual harassment.

The strength of this study lies in its case study design that incorporates use of multiple data sources, which include policy reviews, routine data, and primary qualitative interviews, thereby capturing both quantitative trends and personal lived experiences. It also provides a timely focus on the indirect health effects of pandemic control measures within a fragile health system, emphasizing the disproportionate impact on women and children. Limitations of the quantitative aspect of the study include incomplete routine data (particularly from the DHIS2 database, especially in 2021), and potential underreporting. Limitations with the qualitative findings include selection bias, an urban focus, and the influence of political sensitivity on responses. Additionally, the findings may have limited generalizability beyond the two urban provinces studied, and it is not possible to infer causality due to the absence of statistical testing.

It is difficult to draw firm conclusions about the full impact of COVID-19 and its associated control measures because of the lack of reliable and complete data. Importantly, any future strengthening of the health information system must focus not only on enhancing the ability to conduct infectious disease surveillance and control but also on mitigating negative social, economic, and educational impacts. To improve essential maternal and child health services during lockdowns, specific recommendations include developing protocols to ensure continuity of MNCG services and individual follow-up. This could involve initiatives such as mobile antenatal care (ANC) units, community-based immunization drives, and decentralized antiretroviral therapy (ART) refills. Additionally, pre-identifying vulnerable households for cash or food transfers that can be automatically activated during emergencies would help prevent bureaucratic delays associated with social support in times of crisis. Nonetheless, there are ample data and evidence to suggest that the indirect effects of COVID-19 were at least as harmful, if not more so, than their direct impacts, particularly for women and children.

## Data Availability

The datasets presented in this study can be found in online repositories. The names of the repository/repositories and accession number(s) can be found at: the data that support the findings of this study will be openly available in the London School of Hygiene and Tropical Medicine (LSHTM) at http://doi.org/10.17037/DATA.00004258 (reference number 4258) as soon as the verification process is complete.

## Author’s note

SN was one of the health experts working on the ground who called for lockdowns, as he had first-hand information on healthcare preparedness to face such a novel coronavirus.
