# Supplementary material for: Understanding the impact of the COVID-19 pandemic and its control measures on women and children: a Zimbabwean case study
Source: Front Public Health. 2025 Nov 6;13:1659703. doi: 10.3389/fpubh.2025.1659703 (PMC12631412; doi:10.3389/fpubh.2025.1659703)
Supplement: Supplementary file 1 [file Data_Sheet_1.docx]

**Supplement**

Monthly counts of for each of the indicators were modelled using generalized least squares (GLS) with an autoregressive correlation to account for temporal autocorrelation and a sine and cosine terms for seasonality. Interrupted time series models were specified to estimate both an immediate level change at the onset of the COVID-19 lockdown and a change in slope compared with the pre-COVID-19 trend. Models were estimated using restricted maximum likelihood. Model fit was assessed by Akaike’s Information Criterion and residual diagnostics. Percentage change at the onset of the COVID-19 lockdown was calculated by dividing the estimated level-change coefficient by the predicted counterfactual mean at that time.

Table 1. Interrupted time series analysis for the selected indicators showing the effect of COVID-19 as well as changes in services in the periods before and after the initial COVID-19 lockdowns. The table also shows the percentage change in services due to COVID-19 for each of the indicators.

| **Child growth monitoring visits, Bulawayo**  Percent change after the COVID-19 lockdown: -51.85 (95%CI -71.25 to -32.46) | | | |
| --- | --- | --- | --- |
| **Variable** | **Estimate** | **95%CI** | **p-value** |
| Intercept | 19853 | 17712 to 21995 | <0.001 |
| Pre-COVID-19 slope | -15.87 | -86.46 to 54.73 | 0.655 |
| Level change due to COVID-19 | -9866 | -13557 to -6176 | <0.001 |
| COVID-19 slope-change | 163.41 | -202.91 to 529.74 | 0.376 |
| **Child growth monitoring visits, Harare**  Percent change after the COVID-19 lockdown: -51.86 (95%CI -66.22 to -37.49) | | | |
| **Variable** | **Estimate** | **95%CI** | **p-value** |
| Intercept | 59502 | 56583 to 62421 | <0.001 |
| Pre-COVID-19 slope | -349.42 | -447.03 to -251.81 | <0.001 |
| Level change due to COVID-19 | -21531 | -27496 to -15567 | <0.001 |
| COVID-19 slope-change | -223.59 | -778.24 to 331.07 | 0.423 |
| **Child primary vaccination course complete (PCC), Bulawayo**  Percent change after the COVID-19 lockdown: -2.12% (95%CI -13.50 to 9.25) | | | |
| **Variable** | **Estimate** | **95%CI** | **p-value** |
| Intercept | 1240 | 1171 to 1309 | <0.001 |
| Pre-COVID-19 slope | 0.34 | -1.97 to 2.65 | 0.770 |
| Level change due to COVID-19 | -26.82 | -170.45 to 116.81 | 0.710 |
| COVID-19 slope-change | -9.90 | -23.21 to 3.40 | 0.142 |
| **Child primary vaccination course complete (PCC), Harare**  Percent change after the COVID-19 lockdown: -5.97% (95%CI -25.73 to 13.79) | | | |
| **Variable** | **Estimate** | **95%CI** | **p-value** |
| Intercept | 3426.94 | 3112.93 to 3740.96 | <0.001 |
| Pre-COVID-19 slope | -6.12 | -16.61 to 4.36 | 0.248 |
| Level change due to COVID-19 | -191.04 | -823.20 to 441.12 | 0.548 |
| COVID-19 slope-change | -99.63 | -158.65 to -40.61 | 0.001 |
| **Antenatal care (ANC) 4^th^ visit, Harare**  Percent change after the COVID-19 lockdown: -29.11% (95%CI -70.72 to 12.50) | | | |
| **Variable** | **Estimate** | **95%CI** | **p-value** |
| Intercept | 1605.18 | 956.75 to 2253.61 | <0.001 |
| Level change due to COVID-19 | 8.76 | -12.22 to 29.74 | 0.407 |
| COVID-19 slope-change | -595.62 | -1447.13 to 255.89 | 0.167 |
| Pre-CoVID-19 slope | -96.27 | -194.21 to 1.67 | 0.054 |
| **Antenatal care (ANC) 4^th^ visit, Bulawayo**  Percent change after the COVID-19 lockdown: -33.79 (95%CI -64.52 to -3.07) | | | |
| **Variable** | **Estimate** | **95%CI** | **p-value** |
| Intercept | 371.65 | 180.75 to 562.54 | <0.001 |
| Pre-COVID-19 slope | 10.28 | 4.08 to 16.48 | 0.002 |
| Level change due to COVID-19 | -294.02 | -561.31to -26.72 | 0.032 |
| COVID-19 slope-change | -7.80 | -37.41 to 21.81 | 0.600 |
| **HIV booking visits, Harare**  Percent change after the COVID-19 lockdown: -6.91 (95%CI -55.47 to 41.65) | | | |
| **Variable** | **Estimate** | **95%CI** | **p-value** |
| Intercept | 2924.33 | 1943.31 to 3905.34 | <0.001 |
| Pre-COVID-19 slope | -48.49 | -162.74 to 65.76 | 0.390 |
| Level change due to COVID-19 | -170.35 | -1366.68 to 1025.98 | 0.771 |
| COVID-19 slope-change | 47.97 | -103.46 to 199.40 | 0.520 |
| **HIV booking visits, Bulawayo**  Percent change after the COVID-19 lockdown: -10.53 (95%CI -39.53 to 17.82) | | | |
| **Variable** | **Estimate** | **95%CI** | **p-value** |
| Intercept | 1085.36 | 842.95 to 1327.76 | <0.001 |
| Intercept | 3.83 | -22.43 to 30.09 | 0.766 |
| Pre-COVID-19 slope | -115.37 | -420.13 to 189.38 | 0.443 |
| Level change due to COVID-19 | 5.53 | -31.11 to 42.17 | 0.759 |
